# Refractive Index Sensor Based on Fano Resonances in Metal-Insulator-Metal Waveguides Coupled with Resonators

**DOI:** 10.3390/s17040784

**Published:** 2017-04-06

**Authors:** Yue Tang, Zhidong Zhang, Ruibing Wang, Zhenyin Hai, Chenyang Xue, Wendong Zhang, Shubin Yan

**Affiliations:** 1 Science and Technology on Electronic Test and Measurement Laboratory, North University of China, No. 3 Xueyuan Road, Taiyuan 030051, China; Yue_Tang_1103@163.com (Y.T.); zdzhang@nuc.edu.cn (Z.Z.); bingruiwang_281@163.com (R.W.); xuechenyang@nuc.edu.cn (C.X.); wdzhang@nuc.edu.cn (W.Z.); 2 Ghent University Global Campus, Department of Applied Analytical and Physical Chemistry, Faculty of Bioscience Engineering, 119 Songdo Munhwa-ro, Yeonsu-Gu, Incheon 406-840, Korea; zhenyin.hai@ugent.be

**Keywords:** plasmonic refractive index sensor, finite element method, Fano resonance, coupled-mode theory

## Abstract

A surface plasmon polariton refractive index sensor based on Fano resonances in metal–insulator–metal (MIM) waveguides coupled with rectangular and ring resonators is proposed and numerically investigated using a finite element method. Fano resonances are observed in the transmission spectra, which result from the coupling between the narrow-band spectral response in the ring resonator and the broadband spectral response in the rectangular resonator. Results are analyzed using coupled-mode theory based on transmission line theory. The coupled mode theory is employed to explain the Fano resonance effect, and the analytical result is in good agreement with the simulation result. The results show that with an increase in the refractive index of the fill dielectric material in the slot of the system, the Fano resonance peak exhibits a remarkable red shift, and the highest value of sensitivity (S) is 1125 nm/RIU, RIU means refractive index unit. Furthermore, the coupled MIM waveguide structure can be integrated with other photonic devices at the chip scale. The results can provide a guide for future applications of this structure.

## 1. Introduction

Surface plasmon polaritons (SPPs) are the charge-density waves caused by coupling between photons and electrons on the metal surface [1,2]. Their fields decay exponentially in the direction perpendicular to the metal–dielectric interface [3,4,5,6,7,8]. As a result, SPPs overcome the diffraction limit of light waves [9,10], rendering them suitable for nanoscale photonic devices [11,12,13,14,15]. Therefore, SPPs constitute a new subject that has attracted considerable attention in related fields [16,17], such as biosensing [18,19], SPP lithography [20], and optical and ultrahigh resolution imaging [21].

Among the SPP waveguides, metal–insulator–metal (MIM) waveguides coupled with resonators have flourished and captured the interest of researchers because they can be easily integrated at the chip scale [22,23]. Recently, with the discovery of Fano resonances in plasmonic waveguide structures, the use of plasmonic structures in Fano resonance-based sensors has become increasingly important in many fields, such as physics [24], chemistry [25], biology [26], and energy and information technology [27]. Therefore, many photonic devices based on Fano resonances have been designed by using the coupling effect between narrow dark modes and broad bright modes and have been used in plasmonic sensors [28,29]. However, plasmonic sensors currently present low sensitivity, which remains a huge challenge for researchers.

In this study, a structure composed of MIM waveguides coupled with ring and rectangular resonators is proposed for plasmonic refractive index sensors [30]. A finite-element method (FEM) with perfectly matched layer (PML) absorbing boundary condition is adopted to investigate the properties of the transmission spectra and the refractive index sensing. The magnetic field (H_z_) distributions in this structure are analyzed. In addition, the effects of the structural parameters of the plasmonic coupling system on the Fano resonance are investigated. The function of the shift of the Fano resonance peaks with the refractive index of the fill dielectric is examined.

## 2. Structural Model and Analytical Method

**Figure 1:**
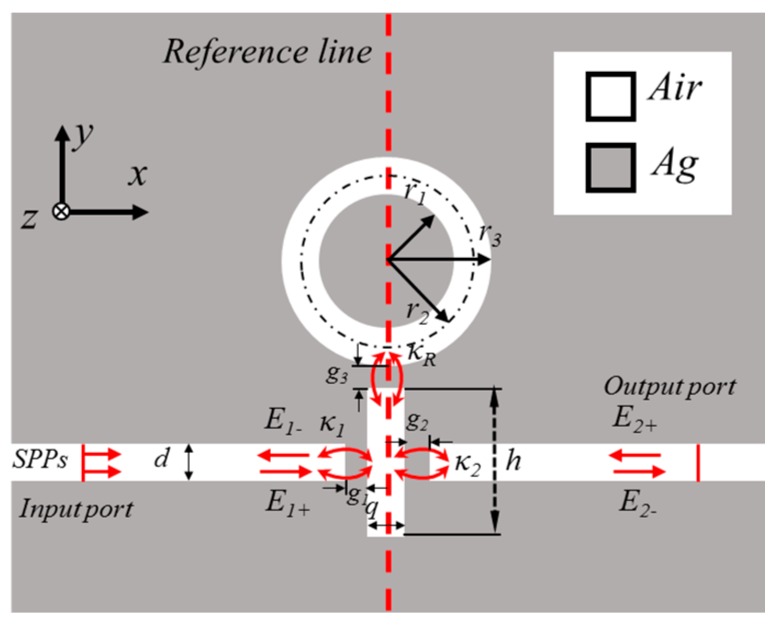
2D schematic of the metal–insulator–metal (MIM) waveguides coupled with rectangular and ring resonators. SPPs: surface plasmon polaritons.

A schematic of the proposed refractive index sensor is shown in Figure 1. The sensor is composed of two MIM waveguides, a rectangular resonator, and a ring resonator. The gray and white areas represent the silver (*ε_m_*) layer and dielectric (*ε_s_*), respectively. The widths of the MIM waveguides, ring cavity, and rectangular cavity are fixed at 50 nm to ensure that only the fundamental transverse magnetic (TM_0_) mode is supported in the MIM waveguides [31]. In Figure 1, *g*_1_, *g*_2_, and *g*_3_ are the coupling distances between the input MIM waveguide and rectangular cavity, between the rectangular cavity and output MIM waveguide, and between the rectangular cavity and ring cavity, respectively. The input and output ports are in the right and left MIM waveguides. The central radius of the ring cavity is *r*_2_ = (*r*_1_ + *r*_3_)/2; *h* and *q* are the height and width of the rectangular cavity, respectively; and *n* is the refractive index of the fill dielectric.

The transmission characteristics of the MIM waveguides coupled with rectangular and ring cavities are simulated by FEM. PMLs were utilized to simulate the top and bottom boundaries of the structure.

The permittivity of Ag can be described by Debye–Drude dispersion mode [31,32]:

(1) $$\varepsilon \left(\omega \right)=\varepsilon_{\infty }+\frac{\left(\varepsilon_{s}-\varepsilon_{\infty }\right)}{\left(1+j\omega \tau \right)}+\sigma /j\omega \varepsilon_{0}$$

where *ε_∞_* = 3.8344 and *ε_s_* = −9530.5 are the infinite frequency permittivity and the static permittivity, respectively; *τ* = 7.35 × 10^−15^ s is the relaxation time; and *σ* = 1.1486 × 10^7^ S/m is the conductivity of Ag.

The TM_0_ model of the MIM waveguide can be expressed as follows [33]:

(2) $$\tan h\left(kd\right)=\frac{-2kp\alpha_{c}}{\left(k^{2}+p^{2}+\alpha_{c}^{2}\right)}$$

where *k = 2π/λ* is the wave vector in the waveguide, *d* is the width of each MIM waveguide, *p = ε_in_/ε_m_* (*ε_in_* and *ε_m_* are the dielectric of the insulator and metal, respectively), and *a_c_ =* [*k*_0_^2^(*ε_in_ − ε_m_*) *+ k*^2^]^1/2^, *k*_0_ is the wave vector in free space. The transmission wavelengths can be derived on the basis of standing wave theory as follows [34,35]:

(3) $$\lambda_{m}=\frac{2Re\left(n_{eff}\right)L}{m-\Psi_{r}/\pi }\left(m=1,2,\cdot \cdot \cdot \right)$$

(4) $$Re\left(n_{eff}\right)=\sqrt{\left[\varepsilon_{m}+{\left(\frac{k}{k_{0}}\right)}^{2}\right]}$$

where *Re*(*n_eff_*)—which is the real part of the effective refractive index of a wavelength in the MIM waveguide—can be derived by Equation (4). In Equations (3) and (4), *L* is the perimeter of the rectangular cavity or ring cavity, *Ψ_r_* is the phase shift of the beam reflected at one end of the cavity.

In this section, the MIM waveguides coupled with the rectangular and ring cavities are analyzed on the basis of temporal coupled-mode theory [36]. To explain the Fano resonance phenomenon, we introduce certain parameters; namely, the SPP wave of the cavity (*E_j_* ± (*j* = 1, 2)) and the coupling coefficients between the input MIM waveguide and rectangular cavity (*κ*_1_), between the rectangular cavity and ring cavity (*κ*_2_), and between the output MIM waveguide and the rectangular cavity (*κ*_3_). When a certain optical wave with *ω* frequency is inputted on the input port of the waveguide (*E*_2+_ = 0), the time evolution amplitudes *A_S_* and *A_R_* of the waveguides of the rectangular and ring cavities, respectively, can be derived as follows [32,37,38]:

(5) $$\frac{\partial A_{s}}{\partial t}=\left(j\omega_{s}-\kappa_{1}^{2}-\kappa_{2}^{2}-\kappa_{R}^{2}\right)A_{s}+\kappa_{1}E_{1+}+\kappa_{2}E_{2+}+\kappa_{R}A_{R}$$

(6) $$\frac{\partial A_{R}}{\partial t}=\left(j\omega_{R}-\kappa_{R}^{2}\right)A_{R}+\kappa_{R}A_{s}$$

where *j* is the imaginary unit (*j*^2^ = −1) and *ω_s_* and *ω_R_* are the resonance frequencies of the rectangular and ring cavities, respectively. In accordance with the energy conservation law, the amplitude of the input and output optical waves in the coupled waveguides are derived by

(7) $$E_{2-}=\kappa_{2}A_{s}$$

Transmittance *T* can be expressed as follows:

(8) $$T={\left|\frac{E_{2-}}{E_{1+}}\right|}^{2}={\left|\frac{\kappa_{1}\kappa_{2}\left[j\left(\omega -\omega_{R}\right)+\kappa_{R}^{2}\right]}{\left[j\left(\omega -\omega_{s}\right)+\kappa_{1}^{2}+\kappa_{2}^{2}+\kappa_{R}^{2}\right]\left[j\left(\omega -\omega_{R}\right)+\kappa_{R}^{2}\right]-\kappa_{R}^{2}}\right|}^{2}$$

where ω is frequency of incident wave and ω = c/λ. When *κ*_1_ = 0.6, *κ*_2_ = 0.24, and *κ*_3_ = 0.14, we get the appropriate fitting curve by the mathematical software.

**Figure 2:**
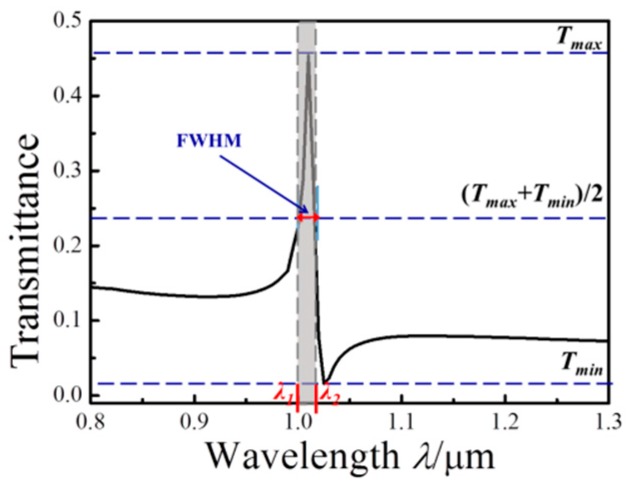
The diagram of full width at half maximum (FWHM).

Additionally, FWHM is full width at half maximum, as shown in Figure 2, which can be expressed as follows:

(9) $$\mathrm{FWHM}=\lambda_{2}-\lambda_{1}$$

where *λ*_1_ and *λ*_2_ are the values on the transmission spectrum at which the transmissivity value is (*T_max_* + *T_min_*)/2. *T_max_* and *T_min_* are the peak and valley values of the transmissivity, respectively. 

## 3. Results and Discussion

**Figure 3:**
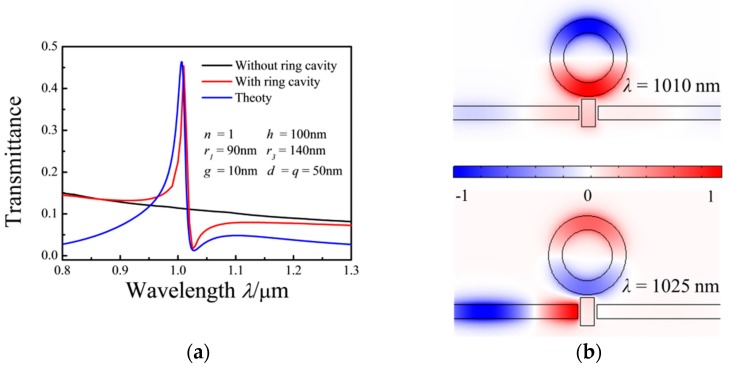
(**a**) Transmission spectra of the MIM waveguides coupled with ring and rectangular cavities (red curve) by a rectangular cavity only (black curve); (**b**) contour profiles of the normalized *H_z_* field distributions in the MIM waveguides coupled with ring and rectangular resonators with *λ* = 1010 nm (top) and *λ* = 1025 nm (bottom).

In this section, the transmission spectra of the MIM waveguides are simulated under varying parameters of the structure. The properties of the transmission spectra can be tuned by these parameters. Figure 3a shows the transmission spectra of the structures with and without a ring cavity. The structure without a ring cavity exhibits low transmittance along a linear curve (black solid curve) with a negative slope. When the MIM waveguides are coupled with both a ring resonator and a rectangular resonator, a peak and a dip exist in the asymmetrical transmission spectrum (red solid curve). The red curve shows a Fano resonance in the MIM waveguide. The blue solid curve is obtained by solving Equation (8), which is in good agreement with the simulation results.

Figure 3b shows the steady-state magnetic field *H_z_* distributions at the points of the peak (*λ* = 1010 nm) and dip (*λ* = 1025 nm) of the MIM waveguides coupled with ring and rectangular cavities. At the peak position (*λ* = 1010 nm), an in-phase relationship exists between the lower parts of the ring resonator and rectangular resonator, whereas an anti-phase relationship is evident between the lower and upper parts of the ring resonator. However, at the dip position (*λ* = 1025 nm), anti-phase relationships exist between the lower parts of the ring resonator and rectangular resonator and between the lower and upper parts of the ring resonator. According to Equations (1)–(4), the effective SPP wavelength, *λ_spp_* = *λ/Re*(*n_eff_*), for *λ* = 1010 nm is 720 nm. At *λ* = 1010 nm, 2π*r*_2_/*λ_spp_* ≈ 1 for the ring resonator, and 2(*h* + *p*)/*λ_spp_* ≈ 0.42 for the rectangular resonator. These results show that *λ_spp_* = 720 nm meets the wave resonance condition of the ring resonator, but does not meet that of the rectangular resonator, which agrees with the numerical results shown in the top image in Figure 3b. Thus, linear and narrow asymmetrical spectra and hybrid and destructive patterns are simultaneously observed between the nonradiative and superradiative modes because of the nonradiative mode and superradiative mode overlap in the spectra.

**Figure 4:**
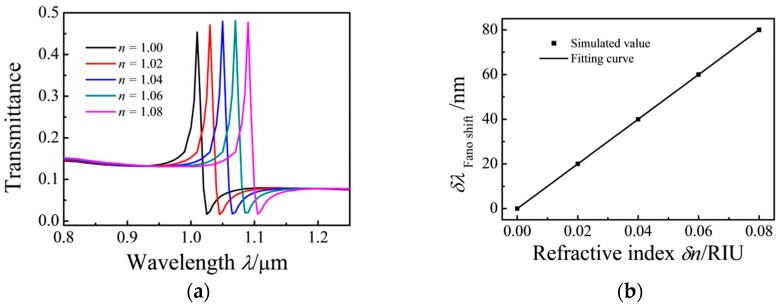
(**a**) Transmission spectra for the MIM waveguide coupled with ring and rectangular cavities with changing *n* (*h* = 100 nm); (**b**) Shift in the Fano resonance peak as a function of refractive index (*δn*).

The transmission spectra are simulated using different filling media to investigate the effect of the refractive index (*n*) on the MIM waveguide. The refractive index, *n*, is increased from 1 to 1.08 at intervals of 0.02 RIU. The simulation results show that the transmission spectrum exhibits a red shift with an increase in *n*. When the effective refractive index *Re*(*n_eff_*) is increased, the Fano resonance peaks demonstrate a red shift with an increase in *n*. Figure 4a shows the shift of the Fano resonance peaks with increasing *n*. The relationship of the peak shift with *δn* is shown in Figure 4b. The sensitivity (S) of the refractive index sensor is *δλ/δn* = 1000 nm/RIU, and the figure of merit (FOM) of the proposed sensor is FOM = S/FWHM = 63 [39].

**Figure 5:**
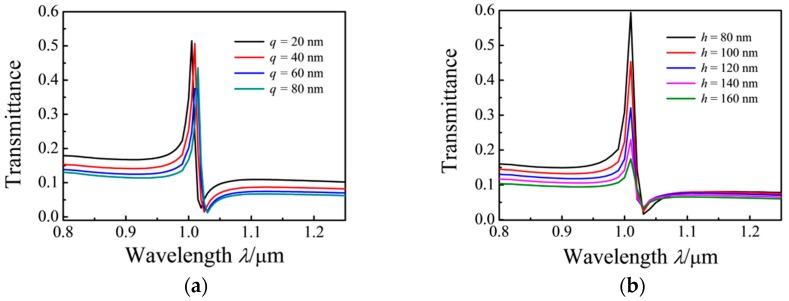
Transmission spectra of the MIM waveguides coupled with ring and rectangular cavities with (**a**) changing *q* (*h* = 100 nm) and (**b**) changing *h* of the rectangular resonator.

For the investigation of the effect of the different widths of the rectangular cavity on the Fano resonance of the MIM waveguide, *q* is varied from 20 nm to 80 nm at intervals of 20 m with *n* = 1, *r*_1_ = 90 nm, *r*_2_ = 115 nm, *r*_3_ = 140 nm, *h* = 100 nm, and *g*_1_ *= g*_2_ *= g*_3_ = 10 nm. The structure of the MIM waveguide is symmetrical about the reference line. With the increasing width of the rectangular cavity, red shifts in the transmission spectrum are observed, as shown in Figure 5a. As the width increases, the value of *L* is increased. This outcome can be explained by Equations (3) and (4). The effect of the height of the rectangular cavity on the transmission characteristics is investigated by varying this parameter from 80 nm to 160 nm at intervals of 20 nm, with *n* = 1, *r*_1_ =90 nm, *r*_2_ = 115 nm, *r*_3_ = 140 nm, *d* = 50 nm, and *g*_1_ *= g*_2_ *= g*_3_ = 10 nm. The spectra show that the transmission rate remarkably decreased with increasing *h*, as shown in Figure 5b. With the increased volume of the rectangular cavity, the light energy confined in the ring cavity and the rectangular cavity is increased.

**Figure 6:**
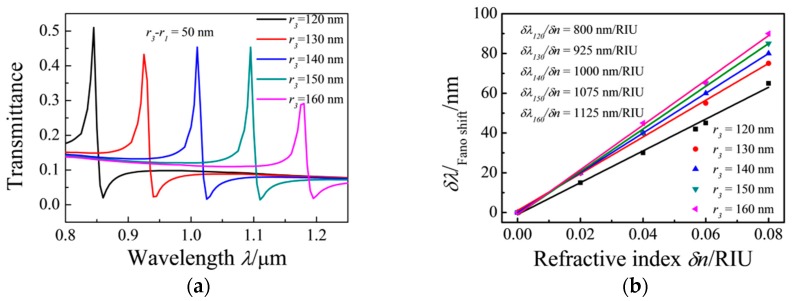
Transmission spectra of the MIM waveguides coupled with ring and rectangular cavities with (**a**) changing *r*_1_ and *r*_3_; (**b**) shift in the Fano resonance peak as a function of the refractive index change (*δn*).

The radius of the ring cavity was changed to study its effect on the transmission rate. The simulation results show that the transmission spectrum exhibits a remarkable red shift with the increasing radius of the ring cavity. The effective refractive index *Re*(*n_eff_*) increases with an increase in the radius of the ring resonator, and the Fano resonance peak exhibits a red shift. *Re*(*n_eff_*) decreases with a decrease in the radius of the ring resonator. Figure 6a shows the shifts in the Fano resonance peaks as *r_1_* and *r*_3_ increase simultaneously. Figure 6b shows the shift in the Fano resonance peak as a function of the refractive index change (*δn*). The results show that with an increase in the radius of the ring cavity, the sensitivity of the MIM waveguide increases from *δλ/δn* = 800 nm/RIU (*r*_3_ = 120 nm) to *δλ/δn* = 1125 (*r*_3_ = 160 nm) and its FOM = 75. Therefore, the sensitivity of the MIM waveguides increases with an increase in the radius of the ring resonator, which causes peak position change [40,41].

**Figure 7:**
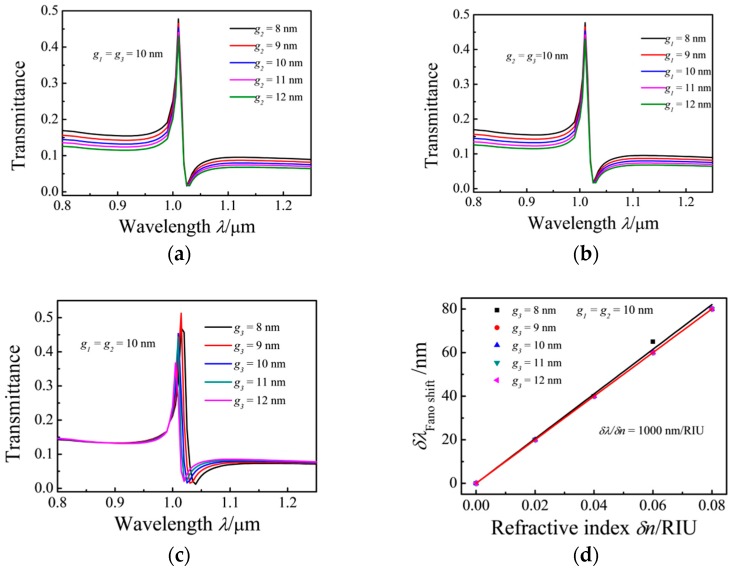
Transmission spectra of the MIM waveguide coupled with ring and rectangular cavities with varying coupling distances (**a**) *g*_2_; (**b**) *g*_1_; (**c**) *g*_3_ and (**d**) the shift in the Fano resonance peak as a function of the refractive index change (*δn*).

Furthermore, the effects of the coupling distances are studied. In the simulation of the effects of coupling distances *g*_1_, *g*_2_, or *g*_3_, the studied parameter is changed while the other two parameters are kept constant. Figure 7a,b show that with the increasing coupling gap between the rectangular resonator and the input MIM waveguide, the energy confined in the structure increases. Figure 7c shows that the transmission spectra exhibit a blue shift as the coupling gap *g*_3_ between the ring cavity and rectangular cavity is increased while the other parameters are fixed at *g*_1_ = *g*_2_ =10 nm, *h* = 50 nm, *d* = 50 nm, *r*_1_ = 90 nm, and *r*_3_ = 140 nm. Figure 7d shows the function of the Fano peak shift with the refractive index change (*δn*); the results show that *δλ/δn* remains constant at 1000 nm/RIU.

## 4. Conclusions

A plasmonic refractive index sensor based on MIM waveguides coupled with rectangular and ring resonators is studied by FEM. The transmission spectra show that the Fano resonance peak that relies on the refractive index of the materials and the perimeter of the ring resonator. With an increase in the ring cavity perimeter and the refractive index, the Fano resonance peak exhibits a red shift. The refractive index sensitivity of the sensor can reach 1125 nm/RIU. In addition, the unit-cell plasmonic structures can be easily integrated with other photonic devices at the chip scale.
